# Circular RNA‐associated ceRNA network involved in HIF‐1 signalling in triple‐negative breast cancer: circ_0047303 as a potential key regulator

**DOI:** 10.1111/jcmm.17066

**Published:** 2021-11-17

**Authors:** Farzaneh Darbeheshti, Mojdeh Mahdiannasser, Zahra Noroozi, Zahra Firoozi, Behnam Mansoori, Abdolreza Daraei, Milad Bastami, Ziba Nariman‐Saleh‐Fam, Elahe Valipour, Yaser Mansoori

**Affiliations:** ^1^ Department of Medical Genetics Fasa University of Medical Sciences Fasa Iran; ^2^ Department of Genetics School of Medicine Tehran University of Medical Sciences Tehran Iran; ^3^ Department of Molecular Medicine School of Advanced Technologies in Medicine Tehran University of Medical Sciences Tehran Iran; ^4^ Department of General Surgery Fasa University of Medical Sciences Fasa Iran; ^5^ Department of Medical Genetics School of Medicine Babol University of Medical Sciences Babol Iran; ^6^ Noncommunicable Diseases Research Center Fasa University of Medical Sciences Fasa Iran; ^7^ Women's Reproductive Health Research Center Tabriz University of Medical Sciences Tabriz Iran

**Keywords:** angiogenesis, ceRNA network, circ_0047303, HIF‐1 signalling, microarray, triple‐negative breast cancer

## Abstract

The aggressive and highly metastatic nature of triple‐negative breast cancer (TNBC) causes patients to suffer from the poor outcome. HIF‐1 signalling pathway is a prominent pathway that contributes to angiogenesis and metastasis progression in tumours. On the contrary, the undeniable importance of circular RNAs (circRNAs) as multifunctional non‐coding RNAs (ncRNAs) has been identified in breast cancer. These ncRNAs owing to their high stability and specificity have been becoming a hotspot in cancer researches. circRNAs act as competing endogenous RNAs (ceRNAs) and compete with mRNAs for shared miRNAs, thus modulate gene expression. Since the most dysregulated biological functions in TNBC are associated with cellular invasion, understanding the molecular pathogenesis of these processes is a crucial step towards the development of new treatment approaches. The purpose of this study is to undermine the circRNA‐associated ceRNA network involved in HIF‐1 signalling in TNBC using an integrative bioinformatics approach. In the next step, the novel circ_0047303‐mediated ceRNA regulatory axes have been extracted and validated across TNBC samples. We show that circ_0047303 has the highest degree in the circRNA‐associated ceRNA network and shows a significant up‐expression in TNBC. Moreover, our results suggest that circ_0047303 could mediate the upregulation of key angiogenesis‐related genes, including *HIF*‐*1*, *EIF4E2* and *VEGFA* in TNBC through sponging the tumour‐suppressive miRNAs. The circ_0047303 could be a promising molecular biomarker and/or therapeutic target for TNBC.

## 1 INTRODUCTION

About 15% of breast cancer (BC) patients are diagnosed with the triple‐negative subtype, which is characterized by an aggressive, highly metastatic entity^1^ and demonstrates the least favourable outcome among all BC subtypes. In triple‐negative breast cancer (TNBC), none of the main receptors (oestrogen receptor and HER2) for BC targeted therapies are expressed, and chemotherapy remains the standard therapeutic choice.^2^ Although TNBC has a high metastasis tendency and a poor outcome, targeted therapy is still limited.^3^ Therefore, it is necessary to seek out potential approaches for targeted therapies. The emergence of high‐throughput techniques such as microarray was accompanied by the identification of BC expression profiles with potential application in diagnosis, prognosis and therapy.^4^, ^5^

In the majority of tumour cells, reduction of oxygen supply (hypoxia) is a common event that evokes a myriad of consequences in tumour cells, namely metastasis, vascularization, epithelial‐to‐mesenchymal transition (EMT) and resistance to therapeutic approaches.^6^ Cellular responses to hypoxia are orchestrated by a group of transcription factors known as hypoxia‐inducible factors (HIFs). These factors trigger the expression of genes essential for adjustment to hypoxic conditions^7^ and affect tumour‐related processes, including metastasis and chemoresistance.^8^ For instance, HIF‐1α impacted metastasis in the MDA‐MB‐435 TNBC cell line.^9^ Expression of HIF‐1α was associated with poor prognosis in BC patients.^10^ In solid tumours, HIF‐1 binds to and upregulates VEGFA under hypoxic conditions. This effect, in turn, promotes the formation of new vessels in tumours.^11^ Altogether, the HIF‐1 signalling pathway is a major driver of angiogenesis and metastasis progression. We previously showed that the most dysregulated biological functions in TNBC are the ones that are involved in proliferation and invasion.^12^ Hence, as TNBC tumours are likely to metastasize, further investigation is needed to better clarify underlying molecular mechanisms in dysregulating HIF‐1 signalling pathway genes.

Years of cancer research provided a great body of evidence that indicates the role of microRNAs (miRNAs) and circular RNAs (circRNAs) in BC (For a complete list of these studies, please refer to [13]). These non‐coding RNAs interact with each other and orchestrate significant processes such as metastasis in cancer cells.^14^, ^15^ One of the functions of circRNAs is to sponge miRNAs, which in turn affects the expression of their target mRNAs.^16^ Such interactions have been investigated in the context of competing endogenous RNA (ceRNA) regulatory networks in BC.^17^, ^18^

In the current study, firstly, we used an integrative approach to determine potential HIF‐1 signalling‐related circRNA‐miRNA‐mRNA axes involved in TNBC tumours using microarray datasets and a variety of bioinformatics tools. In the next step, we sought to underpin and experimentally validate the candidate key differentially expressed (DE) circ_0047303 along with its downstream target genes in triple‐negative tumour samples.

## 2 MATERIALS AND METHODS

### 2.1 Investigation of discriminative differentially expressed circRNAs (DECs) in triple‐negative breast cancer (TNBC) versus normal mammary gland

**FIGURE 1:**
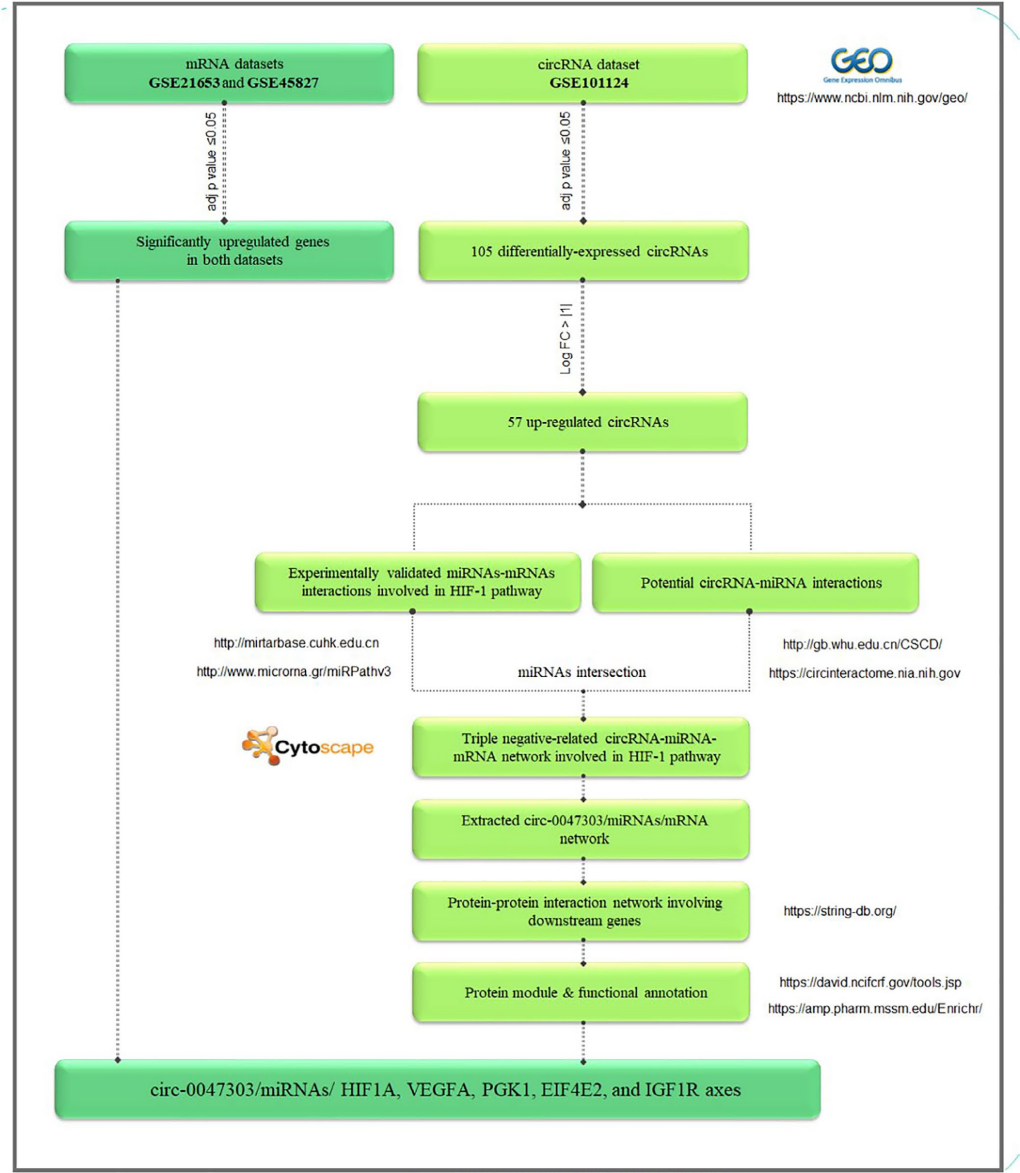
Workflow of in silico study

The microarray dataset GSE101124 related to circRNA expression profile in TNBC containing the data of four triple‐negative tumours and three normal mammary tissues was achieved from the NCBI Gene Expression Omnibus (GEO) database.^19^ The data were normalized (Quantile Normalization) and log2‐transformed (Figure 1). Then to screen the DECs in TNBC in comparison with normal mammary tissues, the Limma package in R software was used. The transcripts with the criteria of |log2(foldchange)| >1 and P‐value <0.05 have been considered as DECs in TNBC. The workflow is briefly shown in Figure 1.

### 2.2 Investigation of differentially expressed genes (DEGs) in triple‐negative breast cancer (TNBC) versus normal mammary gland

Two microarray datasets of GSE21653 and GSE45827 containing protein‐coding genes in both TNBC and normal samples were selected. The former dataset comprised the data of 75 tumour and 29 normal samples and the latter 41 tumour and 11 normal samples. The results were used to select genes for further investigation.

### 2.3 Construction of circRNA/miRNA/mRNA regulatory network involved in HIF‐1 signalling pathway in TNBC

The circRNA/miRNA/mRNA regulatory network involved in the HIF‐1 signalling pathway was constructed according to the following steps:

(1) Prediction of potential interactions between the identified DECs and miRNAs using the CircInteractome database.^20^ (2) The miRNAs involved in HIF‐signalling were identified using DIANA mirPath,^21^ and consequently, their experimentally validated genes were achieved from DIANA TarBase.^22^ Finally, the circRNA/miRNA/mRNA regulatory network was constructed by combining the data obtained in previous steps. Data visualization was performed using Cytoscape.^23^

### 2.4 Construction of protein‐protein interaction (PPI) network and protein modules

STRING database^24^ was used to establish the PPI network consisting of interactions among targets of miRNAs that exist in the constructed circRNA/miRNA/mRNA network. Next, the clustering algorithm ‘Molecular Complex Detection’ (MCODE)^25^ was employed to delineate protein modules. Finally, enrichment analysis and functional annotation were performed using Enrichr and DAVID.^26^

### 2.5 Construction of circ_0047303‐mediated ceRNA network involved in HIF‐1 signalling pathway in TNBC

In the next step, as circ_0047303 has the highest interaction with the miRNAs in the above‐mentioned network, the circ_0047303‐mediated ceRNA subnetwork was selected for further investigation. We hypothesized that some pivotal genes in HIF‐1 pathway could be upregulated through circ_0047303‐mediated miRNA suppression by circ_0047303‐miRNAs‐mRNAs axes. To test our hypothesis, we selected genes that met the following criteria: (a) Targeted by at least two miRNAs in the constructed circ_0047303‐mediated ceRNA subnetwork; (b) underwent an upregulation in TNBC according to microarray datasets GSE21653 and GSE45827; and (c) being involved in the identified protein modules. Thus, five genes, including *HIF*‐*1A*, *VEGFA*, *PGK1*, *EIF4E2* and *IGF1R*, were selected for further validation. First, we evaluated the expression of these genes in TNBC tumours and adjacent normal tissues. Next, we examined the potential correlation between the expression of circ_0047303 and these genes in the samples.

### 2.6 Patients and samples collection

A total of 80 samples, including 40 primary TNBC and 40 matched normal tissues, were obtained from patients referred to Shahid Faghihi Hospital, Shiraz, Iran. None of the patients underwent radiotherapy or chemotherapy before sampling. All individuals have voluntarily participated in the study and have seen and signed the informed consent form. This study has been approved by the ethical committee of Fasa University of Medical Sciences under the ethical ID of IR.FUMS.REC.1398.125.

### 2.7 quantitative real‐time PCR

Extraction of total RNA was performed using TRIzol reagent (Invitrogen) according to the manufacturer's protocol. RNA integrity and quantity were evaluated via gel electrophoresis and spectrophotometry (NanoDrop 2000, Thermo Scientific), respectively. For cDNAs synthesis from total RNA, the PrimeScript™ 1st Strand cDNA Synthesis Kit (TaKaRa Bio, Japan) was used. The sequence of circ_0047303 was obtained from the CircInteractome database, and specific primers were designed and used in real‐time PCR (Table S1). Quantitative real‐time PCR was carried out using RealQ Plus 2x Master Mix Green low Rox (Ampliqon) by Light‐Cycler96 Roche thermocycler. All reactions were performed in 10 µl and duplicated. Gene expression was quantitated using the 2^−ΔΔCt^ method and normalized to the *PUM1* gene as the internal control. PCR products were analysed by melting curve analysis and agarose gel electrophoresis. The circular junction of circ_0047303 was confirmed by Sanger sequencing.

### 2.8 Statistical analysis

Statistical analyses were performed using SPSS software (IBM). Student's *t* test was used to define statistically significant differences. One‐way analysis of variance (ANOVA) was adopted for nonparametric and parametric data. Rock‐curve analysis (ROC) was employed to investigate the diagnostic potential of circRNA biomarker(s). The Kaplan‐Meier method was used to investigate the association of circ_0047303 expression with disease‐free survival (DFS) of patients (data for 30 patients were available during a twenty‐month follow‐up).

## 3 RESULTS

### 3.1 DECs in TNBC versus normal mammary gland tissue based on microarray data

**FIGURE 2:**
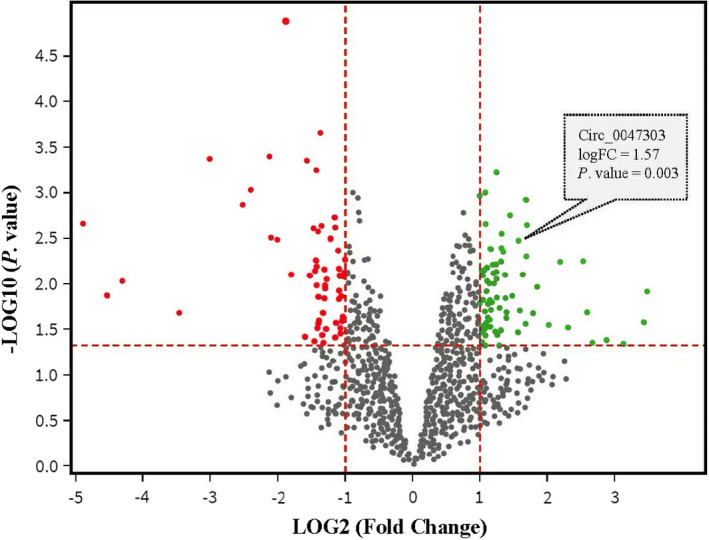
Volcano plot of differentially expressed circRNAs (DECs) in triple‐negative breast cancer (TNBC) compared with normal mammary tissues according to GSE101124. The cut‐off points of fold change (log2 scaled) and *p* value (log10 scaled) are shown by the red dash lines. The red points represent down‐expressed DECs in TNBC, and the green point represents up‐expressed DECs

The GSE101124 dataset was retrieved from GEO and analysed to identify DECs. Comparison of circRNAs expression levels in TNBC versus normal mammary gland tissues revealed 105 DECs, including 57 up‐ and 48 down‐regulated circRNAs (Figure 2 and Table S2).

### 3.2 DEGs in TNBC versus normal mammary gland tissue based on microarray data

The comparison of gene expression levels between TNBC and normal samples from GSE21653 and GSE45827 datasets reveals 2673 and 4123 DEGs, respectively (the data that support these findings are available from the corresponding author upon request).

### 3.3 The circRNA/miRNA/mRNA regulatory network involved in HIF‐1 signalling pathway in TNBC

**FIGURE 3:**
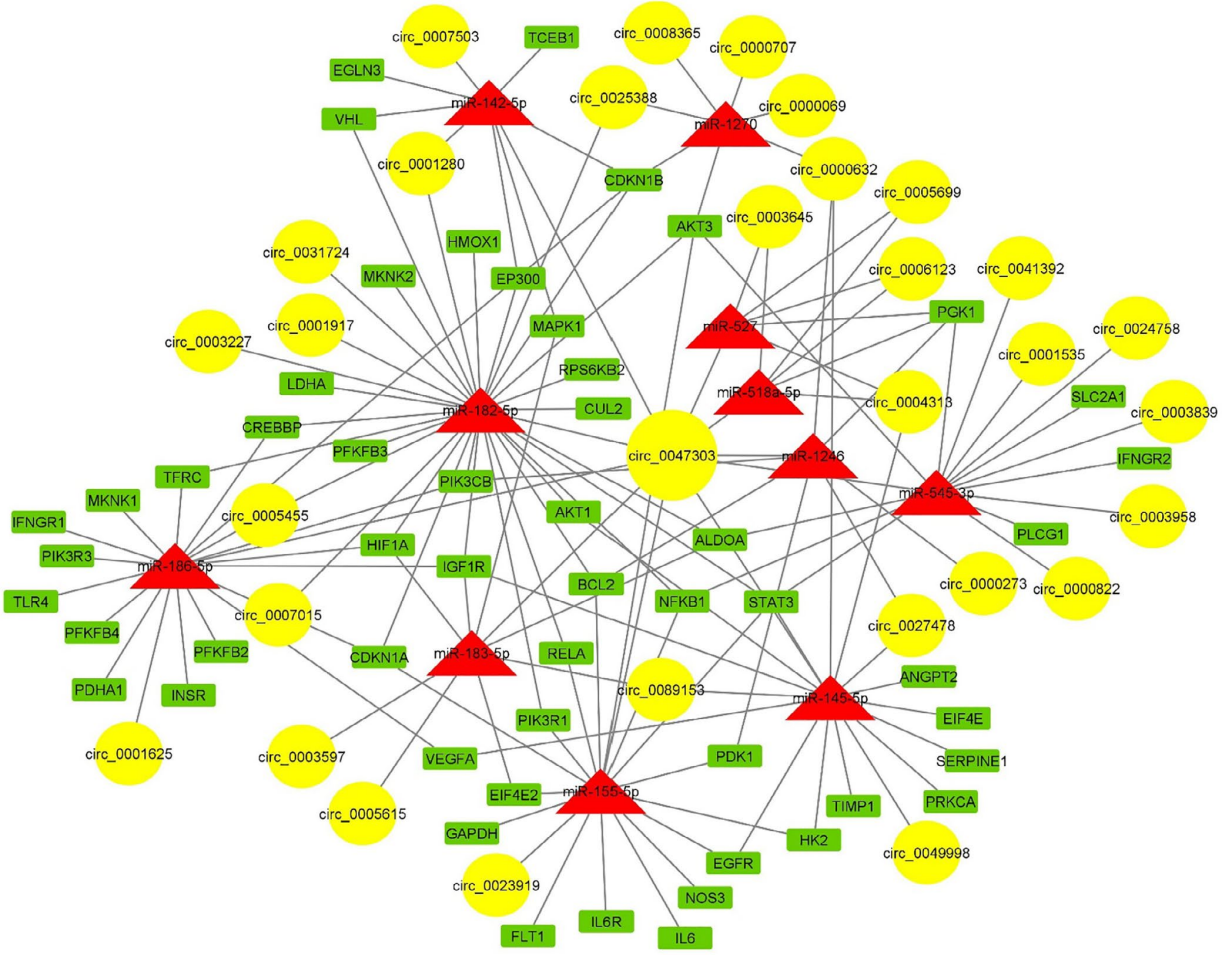
CircRNA/miRNA/mRNA regulatory network involving in the HIF‐1 signalling pathway in TNBC. Yellow circles, red triangles and green rectangles represent circRNAs, miRNAs and mRNAs, respectively. The node size of circRNAs corresponds to their out‐degree

Considering upregulated DECs, the potential circRNA‐miRNA sponging axes involved in HIF‐1 signalling were identified using CircInteractome and DIANA mirPath databases. Eleven miRNAs involved in HIF‐1 signalling pathway were identified that potentially have microRNA response elements (MREs) on sequences of upregulated DECs. Subsequently, 53 mRNAs were uncovered as the experimentally validated targets of the above‐mentioned miRNAs using DIANA TarBase. Finally, the TNBC‐related circRNA/miRNA/mRNA regulatory network involved in HIF‐1 signalling pathway was constructed (Figure 3). This network consists of 68 circRNA‐miRNA pairs and 93 miRNA‐mRNA pairs (Table S3).

### 3.4 The PPI network consisting of downstream targets of the circRNA/miRNA/mRNA regulatory network

Using the STRING database, the PPI network consisting of the downstream genes in the constructed circRNA/miRNA/mRNA network was visualized. This network consisted of 53 nodes and 421 edges (Figure S2A). Next, the protein modules were extracted from the network via MCODE based on densely connected regions of the PPI (Figure S2B). Upon enrichment analysis, it was revealed that these proteins positively regulate cell migration through angiogenesis and negative regulation of apoptosis (Table S4).

### 3.5 Validation of Circ_0047303 Up‐Expression In TNBC compared with tumour‐adjacent normal tissue

**FIGURE 4:**
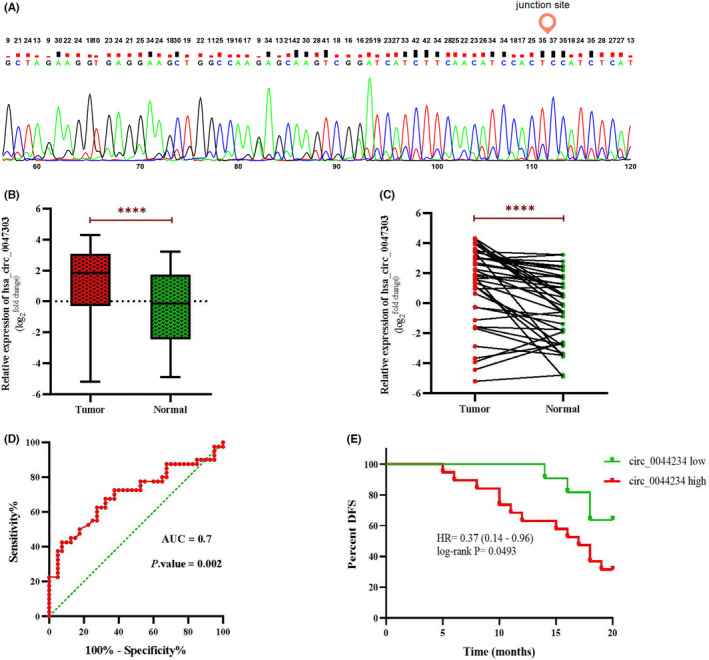
(A) Confirmation of the back‐splice junction site of circ_0047303 by Sanger sequencing. (B) and (C) The expression level of circ_0047303 detected by qRT‐PCR in TNBC samples and matched adjacent normal tissues. (D) Receiver operating characteristic (ROC) curve analysis of the expression of circ_0047303 in TNBC versus normal tissues; AUC, area under the ROC curve. (E) Association of circ_0047303 expression status with disease‐free survival in breast cancer. The red and green lines indicate patients with the high (*N* = 19) and low (*N* = 11) expression level of circ_0047303, respectively

Results of analysing the microarray dataset GSE101124 demonstrated the significant up‐expression of circ_0047303 (hsa_circRNA_102333) in TNBC compared with normal mammary tissues. Here, using quantitative real‐time PCR, the expression of circ_0047303 was evaluated in 40 TNBC and 40 matched adjacent normal tissue samples. Results indicate that circ_0047303 is significantly upregulated in TNBC (Figure 4B and C). The junction site of circ_0047303 was confirmed by sanger sequencing (Figure 4A).

### 3.6 The Circ_0047303 as a potential regulator of key genes in HIF‐1 signalling pathway

**FIGURE 5:**
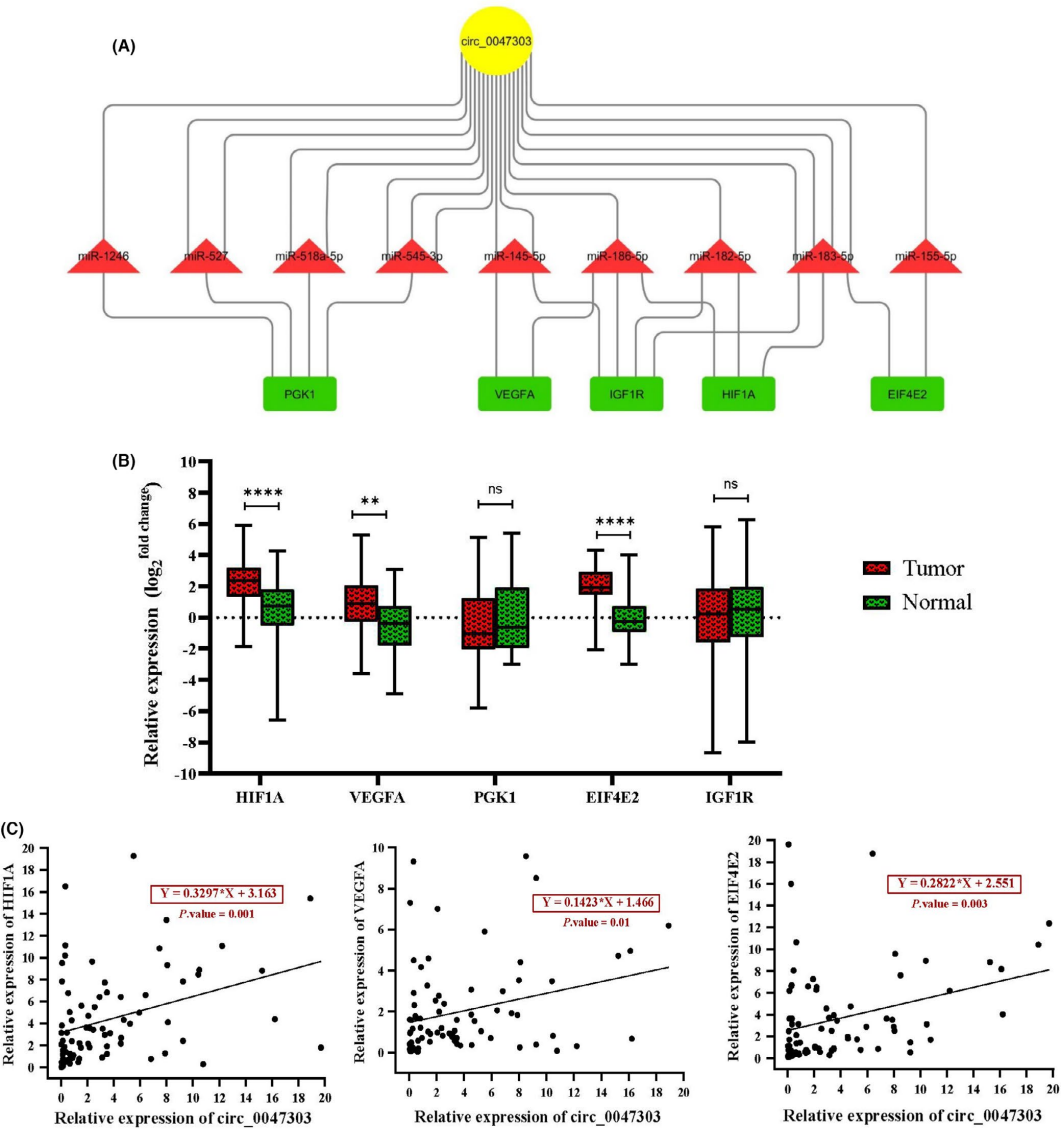
(A) Evaluated circ_0047303‐mediated ceRNA network involved in HIF‐1 signalling pathway in TNBC. The number of edges between circ_0047303 and miRNAs indicates the number of MRE on the circ_0047303 sequence. (B) Expression levels of HIF1A, VEGFA, PGK1, EIF4E2 and IGF1R was detected by qRT‐PCR in TNBC samples and matched adjacent normal tissues. (C) The expression correlation analysis of circ_0047303 with HIF1A, VEGFA and EIF4E2 expression levels in the studied samples. The regression equation has been calculated by linear regression analysis

**FIGURE 6:**
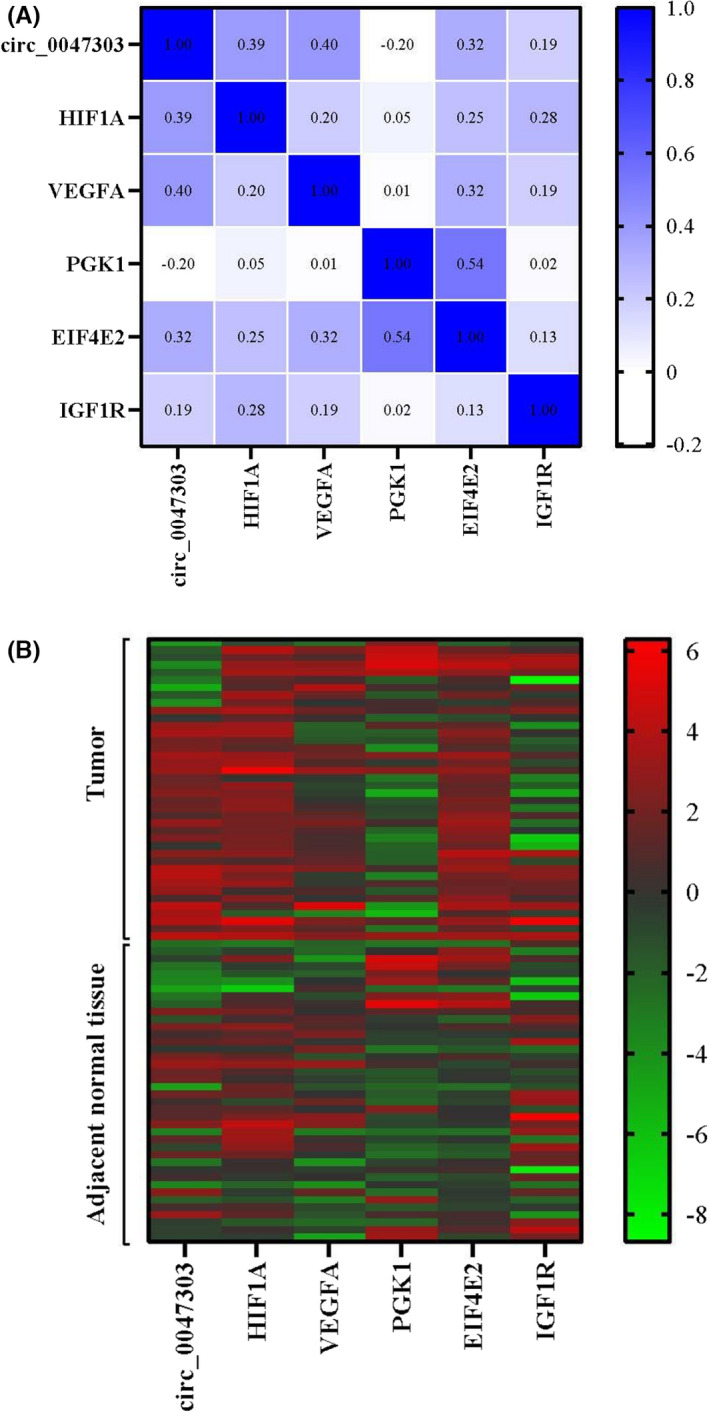
(A) Correlation matrix between circ_0047303 and its downstream gene targets has been represented by a colour scale based on Pearson's correlation coefficient. (B) Hierarchical clustering showing expression values (log2 transformed) of circ_0047303 and its downstream gene targets in TNBC samples and adjacent normal tissues. The expression values are arranged from red (high relative expression) to green (lower relative expression). Each row shows one sample, and each column demonstrates a transcript

The constructed circRNA/miRNA/mRNA network indicates that circ_0047303, the up‐expressed circRNA with the highest degree, could potentially sponge/ inhibit multiple downstream miRNAs that function in the HIF‐1 signalling pathway. According to the criteria which have been formerly explained, the five genes *HIF*‐*1A*, *VEGFA*, *PGK1*, *EIF4E2* and *IGF1R* were selected as downstream targets of circ_0047303‐mediated ceRNA network (Figure 5A). The expression analysis of the five genes by qRT‐PCR reveals that *HIF*‐*1A*, *VEGFA* and *EIF4E2* underwent a significant upregulation in TNBC samples (Figure 5B). Moreover, regression analysis delineated a significant positive correlation between the relative expression of circ_0047303 and that of *HIF*‐*1A*, *VEGFA* and *EIF4E2* genes (Figure 5C). Overall, most Pearson correlation coefficients between circ_0047303 and examined genes were positive (Figure 6A). The heat map from expression data of circ_0047303 and its five downstream genes shows a distinct expression pattern between TNBC and adjacent normal tissues, especially circ_0047303 and *EIF4E2* (Figure 6B).

### 3.7 Clinical implications of circ_0047303 as a diagnostic and prognostic biomarker

The ROC curve results show that circ_0047303 could serve as a potential diagnostic biomarker to discriminate TNBC from normal samples (AUC = 0.7) (Figure 4D). In other words, circ_0047303 can distinguish 70% of patients affected with TNBC with 75% specificity and sensitivity at the optimal cut‐off >1.085. The Kaplan‐Meier method indicates that patients with high circ_0047303 expression have a shorter DFS (Figure 4E). As far as correlation with clinicopathological characteristics of patients, circ_0047303 expression was significantly correlated with tumour size, tumour stage and lymph node metastasis. Therefore, we suggest a prognostic value for this circRNA: expression level of circ_0047303 was positively correlated with tumour size, tumour stage, and risk of metastasis (Table 1).

**TABLE 1:** Association of circ_0047303 expression with clinicopathological features in BC patients

Characteristic	Subgroups	No. of patients (%)	Median	*p*‐value
Age	<50	24 (60)	5.89	0.852
	≥50	16 (40)	5.54	
Tumour size	<2	10 (25)	1.59	**0.021**
	2–5	26 (65)	7.27	
	>5	4 (10)	6.23	
Histologic grade	G1	7 (17.5)	7.54	0.523
	G2	20 (50)	4.81	
	G3	13 (32.5)	6.22	
TNM stage^a^	I	8 (21)	1.01	**0.0001**
	II	17 (44.7)	3.55	
	III	11 (29)	11.24	
	IV	2 (5.3)	13.3	
Lymph nodes metastasis	Yes	18 (45)	8.02	**0.003**
	No	22 (55)	2.05	
Histologic type of invasive carcinoma	IDC	35 (87.5)	5.9	0.96
	ILC	5 (12.5)	4.69	

Abbreviations: IDC, invasive ductal carcinoma; ILC, invasive lobular carcinoma.

The significant associations are shown in bold.

^a^ Two patients information missing.

## 4 DISCUSSION

For the first time, this study has introduced the involvement of circ_0047303/miRNA/mRNA ceRNA network in triple‐negative breast cancer via HIF‐1 signalling. Our results demonstrated remarkably enhanced levels of circ_0047303 and three angiogenesis‐associated genes including *HIF*‐*1*, *EIF4E2* and *VEGFA* in triple‐negative samples. Furthermore, our data suggest that circ_0047303 could serve as a potential diagnostic (AUC = 0.7) and prognostic biomarker for TNBC. Our study was on the most aggressive BC subtype, which is associated with a poor outcome and the highest metastasis rate compared to other subtypes,^27^, ^28^ and leads to rapid deterioration and distant metastasis in patients.^29^

We looked up potential DE circRNAs that underly TNBC progression through their function in HIF‐1 signalling pathway. These RNAs harbour MREs that bind to and sponge specific miRNAs and regulate the expression of their target genes.^30^, ^31^

Results of microarray data analysis showed that circ_0047303 was significantly upregulated in TNBC compared to normal mammary tissues. Moreover, we found circ_0047303 shows the highest degree in the constructed circRNA/miRNA/mRNA network (Figure 3). According to the selection criteria adopted in this study, the downstream genes of circ_0047303‐mediated ceRNA network should be: (i) targeted by at least two miRNAs predicted to be sponged by circ_0047303; (ii) upregulated in TNBC according to microarray data analysis results; and (iii) involved in the identified protein modules. Five HIF‐1 signalling‐related genes, including *HIF*‐*1A*, *VEGFA*, *PGK1*, *EIF4E2* and *IGF1R*, were determined as downstream targets of circ_0047303‐mediated ceRNA axes (Figure 5A). We assume that these genes could be involved in the circ_0047303/miRNA/mRNA network and that in the case of circ_0047303 upregulation, miRNAs are sponged away from target mRNAs and can no longer inhibit their expression. Thus, it might explain the upregulation of their target genes.

Our experimental results indicate that circ_0047303, also known as hsa_circRNA_102333, is significantly upregulated in TNBC compared with adjacent normal tissues, further confirming the findings of microarray data analysis. Moreover, we found that the expression signature of circ_0047303 has a diagnostic value for TNBC with AUC 0.7 at the optimal cut‐off >1.085 (75% specificity and sensitivity). We also examined the prognostic value of circ_0044234 expression in terms of DFS in TNBC patients. The data show that the high expression level of circ_0047303 in the tumour samples tends to lower DFS (*p* = 0.049). As the follow‐up time in the current study is short (20 months), it is suggested that the prognostic value of circ_0047303 be investigated in a larger sample size and a longer follow‐up time in future researches.

Additionally, association analysis of the patients’ clinicopathological features and the expression level of circ_0047303 revealed a significant positive correlation between the expression of circ_0047303 and tumour size, lymph node metastasis, and TNM stage. Higher TNM stage and larger tumour size were correlated with higher expression of circ_0047303. Besides, tumours with lymph node metastasis had a higher expression rate compared to non‐metastatic tumours. In conclusion, we suggest that circ_0047303 could serve as a novel potential diagnostic and prognostic biomarker in TNBC. The circ_0047303 is an exonic circRNA derived from exon 3 of *ZNF521* gene located at chromosome 18. Upregulation of circ_0047303 has also been reported in hepatocellular carcinoma.^32^ However, the current knowledge of the oncogenic role of this circRNA in different malignancies is relatively limited.

In the following step, the expression of five downstream genes in circ_0047303‐mediated ceRNA axes (Figure 5a) was evaluated by qRT‐PCR. The results show a significant upregulation of *HIF*‐*1A*, *VEGFA* and *EIF4E2* genes in TNBC (Figure 5b).

It has been shown that *HIF*‐*1* is involved in tumorigenesis and/or tumour progression: overexpression of *HIF*‐*1α* or *HIF*‐*2α* was linked with mortality in several cancers, including BC.^33^ Currently, inhibition of *HIF*‐*1* is considered as a cancer therapy approach, and many drugs have been developed that serve this purpose.^33^ Given that HIF‐1 is a crucial transcription factor to *VEGFA* expression,^34^ the drug‐mediated lack of HIF‐1 might also explain the mechanism underlying decreased VEGFA expression. High plasma levels of VEGFA, an important angiogenic factor, had a positive correlation with advanced tumour stages in BC patients.^35^ Similarly, Roberti, et al. demonstrated that VEGFA protein expression is a potential prognostic biomarker for TNBC.^36^ It has been recently shown that VEGF signalling exerts immune suppressive effects in BC tumours.^37^ In hypoxia, the eukaryotic initiation factor 4E type 2 (EIF4E2*)* embarks on the translation of HIF‐2α‐bound mRNAs that contain hypoxia response elements.^38^ Silencing of *EIF4E2* in MDA‐MB‐231 triple‐negative cell line indicated a remarkable decrease in tumour cell migration and invasion during hypoxia.^38^

Our data suggest a significant positive correlation between the expression of circ_0047303 and that of *HIF*‐*1A*, *VEGFA* and *EIF4E2* genes (Figure 5C). Interestingly, circ_0047303 harbours four MREs for the seed region of miR‐183‐5p (Figure 5A), which could silence both *HIF*‐*1A* and *EIF4E2* genes. Therefore, we suggest that circ_0047303 might modulate the expression of these genes in hypoxia, and the upregulation of this circRNA results in the up‐expression of *HIF*‐*1A* and *EIF4E2* in TNBC through decreasing the intracellular levels of miR‐183‐5p.

There are contradictory data regarding the dysregulation and function of miR‐183 in BC. In line with our hypothesis, some studies have documented its tumour‐suppressive role. For instance, upregulation of miR‐183 prevents migration and invasion of breast tumours.^39^ Recently, Anderson, et al. reported significant down‐expression of miR‐183‐5p in post‐EMT (epithelial‐mesenchymal transition) breast cancer cells. Moreover, restoring the expression of miR‐183‐5p with an exogenous mimic resulted in a significant decrease in BC cell invasion and migration.^40^ In contrast, several studies revealed the upregulation and oncogenic role of miR‐183‐5p in BC.^41^, ^42^ These inconsistent observations could be caused by different expression profiles between various intrinsic BC subtypes. Here, for the first time, we propose the potential role of circ_0047303/ mir‐183‐5p/ HIF‐1A and EIF4E2 regulatory axes in TNBC pathogenesis according to our experimental and *in silico* investigation.

Another target of circ_0047303 is miR‐145 for which it harbours two MREs (Figure 5A). Regarding direct suppression of VEGFA by miR‐145,^43^ we suggest that up‐expressed circ_0047303 could modulate the expression of VEGFA through sponging miR‐145 in TNBC. Various studies have shown the downregulation of miR‐145 in BC^44^ and TNBC tissues and cell lines.^45^, ^46^ There is evidence that miR‐145 upregulation in BC exerts a tumour repressive effect through inhibition of angiogenesis and cell invasion.^43^ Therefore, miR‐145 has been previously suggested to act as a tumour suppressor, and in the current study, we have uncovered a potential underlying mechanism to suppress miR‐145‐5p in TNBC: up‐expression of circ_0047303 as a sponge! Altogether, we propose the TNBC‐related ceRNA axes comprising circ_0047303/miR‐183‐5p/HIF‐1 and EIF4E2 as well as circ_0047303/miR‐145‐5p/VEGFA.

Although *in silico* findings in this study were experimentally validated, a certain shortcoming here is the lack of in vivo/in vitro approaches such as animal modelling and cell culturing, respectively. Such studies provide functional evaluation of circ_0047303 and further facilitate understanding the potential role of circ_0047303 and molecular interactions within the circRNA/miRNA/mRNA network in angiogenesis through HIF‐signalling pathway.

In conclusion, we used an integrative systems biology‐directed strategy to study the molecular mechanism underlying angiogenesis in TNBC. By combining microarray datasets and using bioinformatics tools, we constructed a circRNA/miRNA/mRNA network involved in HIF‐1 signalling pathway in TNBC. We found circ_0047303 as the highest degree centrality node in this ceRNA network, which showed a significant up‐expression in triple‐negative tumours. Our data indicate that circ_0047303 has a potential value as a diagnostic and prognostic biomarker in TNBC. In the next step, we delineated the novel circ_0047303‐mediated ceRNA axes involved in the aggressive behaviour of TNBC using an *in silico*‐*in vitro* approach. Our data suggest that upregulation of three angiogenesis‐associated genes including *HIF*‐*1*, *EIF4E2* and *VEGFA* in TNBC could be mediated by circ_0047303 through sponging the tumour‐suppressive miRNAs. This study, for the first time, shines a light on the role of circRNAs in HIF‐1 signalling pathway in TNBC and suggests that circ_0047303 is a promising molecular biomarker and/or therapeutic target. Nevertheless, future studies are required to examine the possible clinical applications.

## CONFLICTS OF INTEREST

The authors declare no conflict of interest.

## AUTHORS CONTRIBUTIONS

F.D. and Y.M.: Conceptualization. F.D.: Methodology. F.D. and A.D.: Validation. F.D. and M.B.: Formal analysis. F.D., Z.N. and Z.F.: Investigation. Y.M and B.M.: Resources. F.D. and M.M.: Writing‐original draft preparation. F.D., Y.M. and Z.N.S.F.: Writing‐review and editing. F.D., M.M. and E.V.: Visualization. Y.M.: Funding acquisition. All authors have read and agreed to the published version of the manuscript.

## Supporting information

Fig S1‐S2

Table S1‐S4

## Data Availability

The data that support the findings of this study are available in the supplementary material of this article. Moreover, the analysed data of two GEO datasets GSE21653 and GSE45827 are available from the corresponding author upon request.
