# Supplementary figures and images for: Circular RNA‐associated ceRNA network involved in HIF‐1 signalling in triple‐negative breast cancer: circ_0047303 as a potential key regulator
Source: J Cell Mol Med. 2021 Nov 17;25(24):11322–32. doi: 10.1111/jcmm.17066 (PMC8650046; doi:10.1111/jcmm.17066)

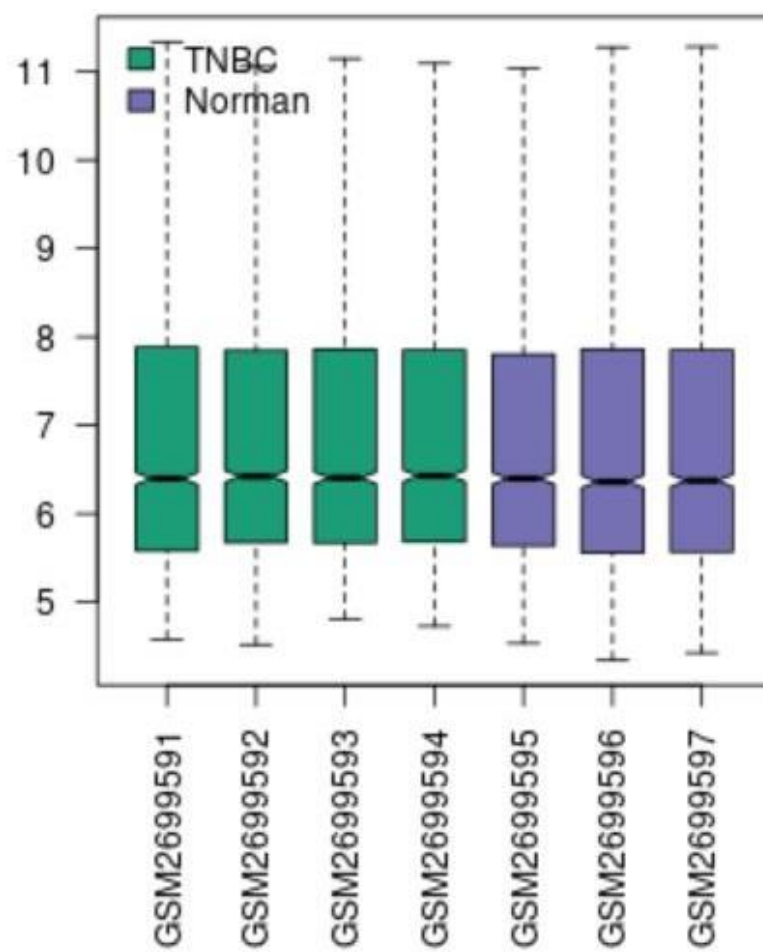

Supplementary Figure 1.

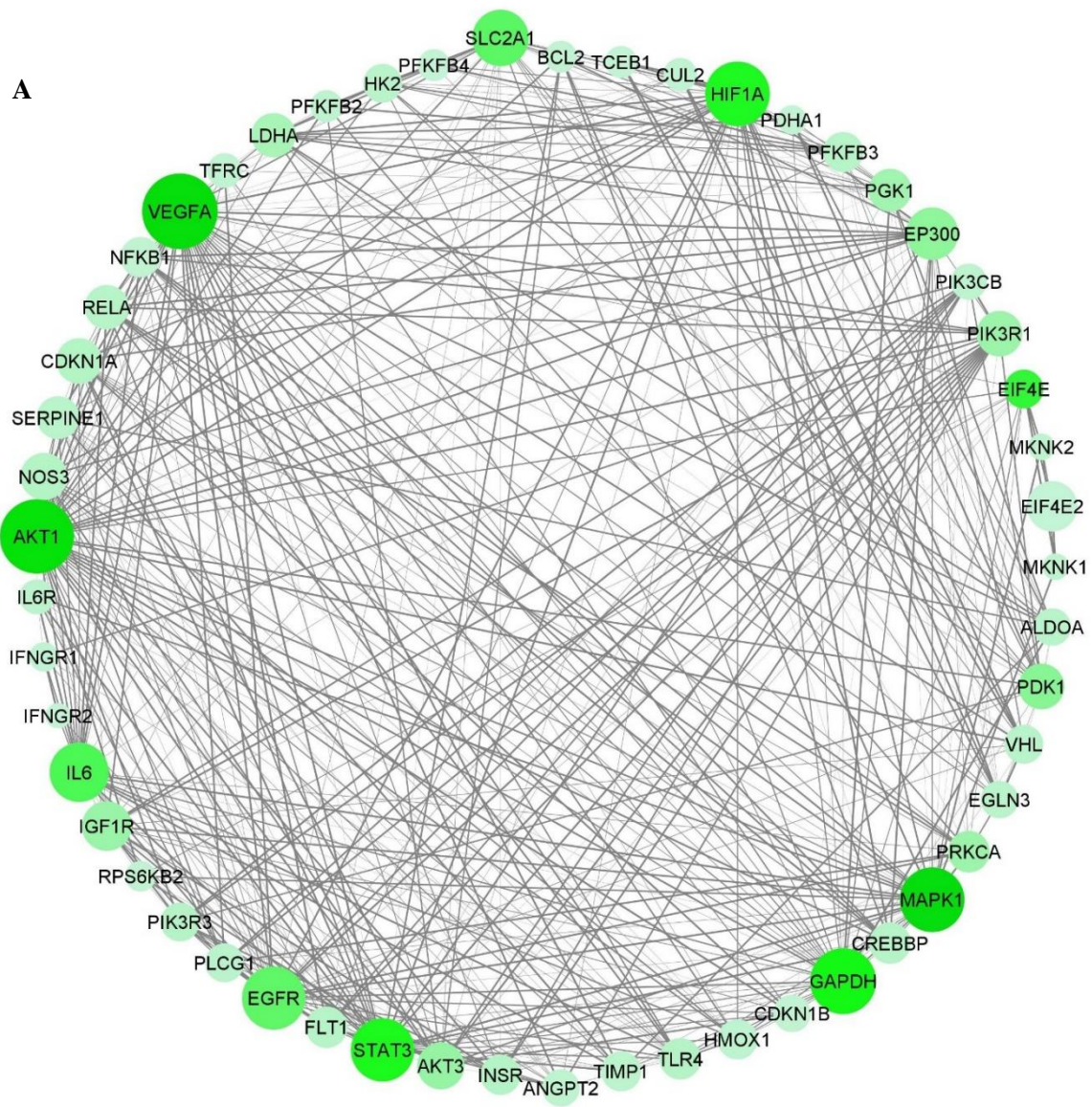

**B**

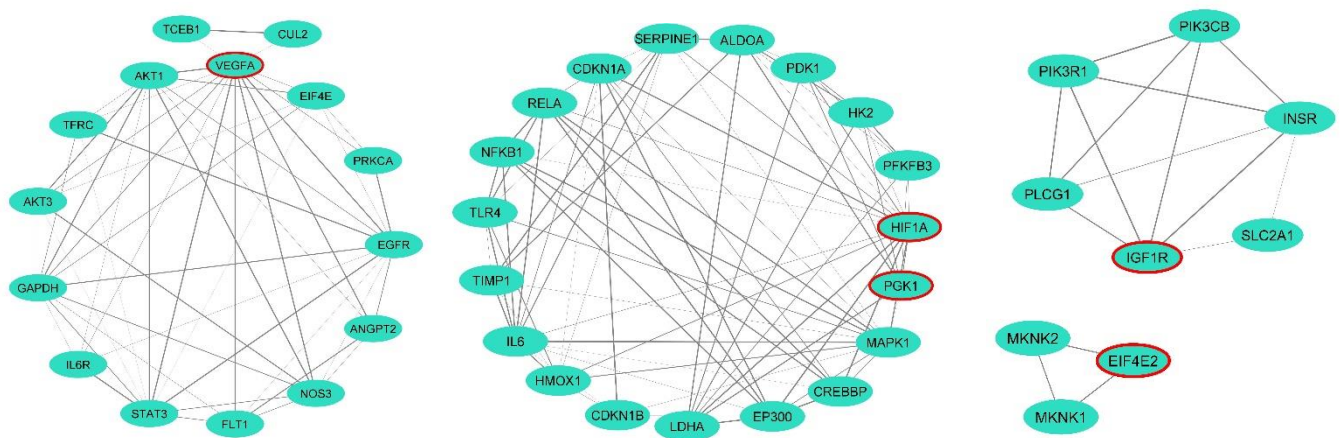

**Supplementary Figure 2.**

Supplement: Supplementary file 1 — Fig S1‐S2 [file JCMM-25-11322-s001.pdf]
